# The Legal Guarantee for Achieving Carbon Peak and Neutrality Goals in China

**DOI:** 10.3390/ijerph20032555

**Published:** 2023-01-31

**Authors:** Hui Shi, Xiaobin He

**Affiliations:** 1 School of Law, Qingdao University, Qingdao 266071, China; 2 School of Law, Shandong University, Qingdao 266237, China

**Keywords:** carbon neutrality, carbon peak, legal guarantee, 1 + N policy system, Chinese characteristics, central and local initiative

## Abstract

In order to actively fulfill its international treaty obligations, China has established the goal of peaking CO_2_ emissions by 2030 and achieving carbon neutrality by 2060. Since 2018, when ecological civilization was written into the Constitution, the realization of carbon peak and neutrality goals has had an ideological foundation and a constitutional basis. China has formulated various special laws and built a 1 + N policy system to reduce carbon emissions, which together with the environmental protection law, climate change law, energy law and other related laws and regulations constitute a unified legal system and provide legal support to achieve carbon peak and neutrality goals. At the same time, China has taken advantage of the new national system with concentrated efforts and resources to delineate the different roles of the government and market mechanisms in carbon emission reduction, and to make the operation of the legal system of carbon peak and neutrality suitable for its actual situation by giving full paly to the initiative of both central and local governments. This article analyzes the current legal system and its characteristics in China in the process of achieving carbon peak and neutrality goals in the context of the new era, and outlooks on the improvement path of the legal system from both domestic and international dimensions. The practice, experience and development direction of China in the construction of the legal guarantee for carbon peak and neutrality goals can provide reference for other countries to achieve carbon reduction.

## 1. Introduction

In October 2018, the United Nations Intergovernmental Panel on Climate Change released a special report: Global Warming of 1.5 °C. The report states, in conjunction with Article 2 of the Paris Agreement, that global net artificial CO_2_ emissions will reach net zero around 2050 in the 1.5 °C model. In contrast, in the 2 °C model, net zero will be reached around 2050 [1]. Net-zero emissions refer to carbon neutrality, meaning that global CO_2_ emissions will be balanced over a certain period of time through the elimination of artificial CO_2_ emissions. In terms of global progress towards the carbon neutrality goal, as of September 2022, 17 countries have enacted relevant laws, 38 countries have enacted relevant policies, 16 countries have made relevant commitments and 57 countries are still in the process of discussion [2].

As an important promoter for the international governance of climate change, China has made important contributions to the process of promoting the construction of international conventions and treaties frameworks. China ratified the United Nations Framework Convention on Climate Change in 1992 and signed the Kyoto Protocol in 1998. Since the Copenhagen Climate Change Conference in 2009, China has become more proactive in integrating into the international climate change governance framework. In 2012, China and other developing countries facilitated the adoption of the Doha Amendment to the Kyoto Protocol. From 2014 to 2015, China issued joint statements with the United States, France and the United Kingdom, playing an important role in the conclusion and implementation of the Paris Agreement. The above-mentioned international treaties established a platform for action by all countries on the issue of controlling carbon emissions. On 22 September 2020, President Xi Jinping announced at the 75th session of the United Nations General Assembly that China strives to peak CO_2_ emissions by 2030 and achieve carbon neutrality by 2060.

The proposed long-term climate goal of achieving net-zero greenhouse gas emissions by 2060 has sparked widespread debate at home and abroad. The vigorous promotion of achieving carbon neutrality is not only a major strategic decision to respond to global climate change and promote the construction of a global environmental governance system, but also a key initiative to promote the construction of ecological civilization and build a community with a shared future for mankind. To achieve the ambitious goal of carbon neutrality, China has begun to structure a technological inspiration system in coordinating and facilitating scientific and technological research and innovations. First and foremost, China should move away from fossil fuel dependence, increase the share of clean energy and reduce total greenhouse gas emissions, which is foreseeable with extensive application of coal-based clean fuel and chemical technology, new energy generation technology (e.g., hydrogen energy, nuclear energy and solar energy), renewable energy non-electric utilization technology, etc. In addition, CCUS (carbon capture, utilization and storage) measures, such as carbon-negative technology, carbon sink monitoring technology, ecosystem carbon fixation technology and non-carbon dioxide greenhouse gas replacement technology, should also be stepped up to offset the total amount of greenhouse gases already emitted [3].

There is general agreement that in order to promote the orderly realization of the goal of carbon neutrality, the legal system should be strengthened and guaranteed through the rule of law, and scholars at home and abroad have carried out corresponding research. Some scholars discuss the international conventions and declarations signed by China and the implementation in the context of greenhouse gas emission trends [4]. Others investigate the construction and operational effectiveness of the legal system in the process of carbon peak and neutrality goals from the two major objectives of promoting carbon emission reduction and achieving sustainable economic and social development [5], and focus on the construction and operational effectiveness of the legal system of the carbon-trading market in China [6]. It is worth noting that, at this stage, the current legal system for achieving carbon peak and neutrality goals has taken on new characteristics of the times and development needs. During the 14th Five-Year Plan period (2021–2025), China is pressing ahead with the implementation of a series of relevant regulations and policies to promote carbon peak by 2030 and to provide the preconditions and guarantees for achieving carbon neutrality by 2060.

This article analyzes the current legal system and its characteristics in the process of achieving carbon peak and neutrality goals in the context of the new era, as well as outlooks on the improvement path of this legal system from both domestic and international dimensions. It argues that China must effectively fulfill its international treaty obligations and responsibilities based on its economic, political, social and cultural development, and comprehensively promote the construction and improvement of the legal system to achieve carbon peak and neutrality. At the same time, efforts should be made to build a new type of national system to combine the role of the government and the market mechanism, so as to mobilize the enthusiasm of local governments under the unified leadership of the central government and provide a solid legal guarantee for the orderly realization of carbon peak and neutrality goals.

## 2. Current Legal System to Achieve Carbon Peak and Neutrality Goals in China

On the basis of the recognition of the importance of the rule of law to the modernization of national governance, China has focused on constructing a legal system to achieve carbon peak and neutrality goals. Up to now, China has formed a legal system with the Constitution as the guideline, special laws and policies for carbon peak and carbon neutrality as the core and other related laws, such as environmental protection law, climate change law and energy law, as the framework.

### 2.1. Constitutional Basis for Carbon Peak and Neutrality

Since 1978, the Constitution in China has explicitly stated that the country shall protect the environment and natural resources, and prevent and control pollution and other public hazards. In 1982, when the Constitution was amended, environmental protection was directly established as a basic state policy. After the adoption of the 2018 amendment, the preamble of the Constitution explicitly calls for implementing the new concept of development and promoting the coordinated development of ecological civilization and material, political, spiritual and social civilization. The inclusion of ecological civilization in the Constitution demonstrates the importance of ecological civilization in the national development strategy in the form of a fundamental law, and environmental protection is no longer dependent on the development of economic, political or other social areas, but has become an independent national goal. The content of ecological civilization in the Constitution also provides the ideological basis and value guidance for the construction and development of the legal system for carbon peak and neutrality goals in China [7].

Apart from that, some specific provisions in the Constitution are closely related to environmental protection, providing a constitutional basis for the construction of the legal system for carbon peak and neutrality goals. Article 9 and Article 26 of the Constitution require safeguarding the rational utilization of natural resources and preventing pollution and other public hazards. An important way to achieve carbon peak and neutrality goals is to promote industrial upgrading, improve energy structure, reduce energy consumption, further promote rational utilization of energy, stop energy waste and reduce greenhouse gas emissions. Article 26 and Article 89 call for the protection and improvement of the living environment and ecological environment. This is in line with the preamble of the Constitution, which stipulates the goal of implementing the new development concept and promoting the coordinated development of ecological civilization and other civilizations. The climate change caused by excessive greenhouse gas emissions is closely related to the living environment and ecological environment, and it will profoundly affect the process of building ecological civilization. Article 89 empowers the State Council to lead and manage the construction of ecological civilization, which provides the constitutional basis for the State Council and related departments to promote the achievement of carbon peak and neutrality goals through administrative regulations, departmental rules and normative documents.

### 2.2. Specialized Legal and Policy System for Carbon Peak and Neutrality Goals

To ensure the smooth achievement of carbon peak and neutrality goals, China adheres to the rule of law, elaborates top-level design to maximize the institutional advantages of the legal system, as well as facilitates and motivates the government sectors to formulate policies according to actual conditions, and a special law and 1 + N policy system has been established.

#### 2.2.1. Specialized Legal Norms for Carbon Peak and Neutrality Goals

The specialized legal norms for carbon peak and neutrality goals focus on the trading and management order of the carbon emission market. Practice shows that improving the trading and management of the carbon emission market through the rule of law is an important means to control greenhouse gas emissions, promote low-carbon development and achieve energy conservation and emission reduction, as well as a vital way to achieve carbon peak and neutrality goals [8]. After carbon peak and neutrality goals were proposed, two departmental regulations were formulated by the constituent departments of the State Council to address the trading and management issues in the carbon emission market.

In 2020, the Ministry of Ecology and Environment promulgated the Measures for the Administration of Carbon Emission Trading (for Trial Implementation). The management measures are the first legal regulation in the nature of departmental regulations applicable to trading and related activities for national carbon emissions after carbon peak and neutrality goals were proposed. According to the regulation, the trading product of the national carbon emission market is carbon emission allowances, whose total amount is determined and allocated by the Ministry of Ecology and Environment and allocated by the provincial ecological and environmental authorities to the key emission units within the administrative region. The trading subjects of the national carbon emission trading market mainly refer to the units belonging to the industries covered by the national carbon emission trading market or whose annual greenhouse gas emissions reach 26,000 tons of carbon dioxide equivalent. The competent department of ecology and environment is responsible for supervising, inspecting and guiding the national carbon emission trading activities, and the staff and key emission units who violate the measures shall be punished and penalized according to the law, and those who are involved in crimes shall be sent to judicial organs. The Interim Regulations on the Management of Carbon Emission Trading (Draft Revision), drafted by the Ministry of Ecology and Environment in 2021, further clarify the basic principles and division of responsibilities for the management of carbon emission trading and refine the total amount of carbon emission allowances, allocation methods, as well as the rights and obligations of carbon emission subjects.

From the development history of carbon trading mechanisms in various countries, the construction of carbon trading mechanisms usually involves multiple actors at home and abroad, and is affected by various economic and trade factors, so it needs to be continuously improved and strengthened over a long period of time [9]. The above-mentioned departmental regulations on national carbon emission trading and related activities initially constructed a legal system to provide a legal guarantee for binding national carbon trading market activities.

#### 2.2.2. The 1 + N Policy System for Carbon Peak and Neutrality Goals

In response to the domestic and international energy market situation and economic development, China proposed the 1 + N policy system for carbon peak and neutrality goals in 2021. In September 2021, the Central Committee of the Communist Party of China and the State Council issued the Opinions on the Complete and Accurate Implementation of the New Development Concept to Achieve Carbon Peak and Neutrality Goals. This is the programmatic document in the 1 + N policy system for carbon peak and neutrality goals. It proposes the promotion the comprehensive green transformation of economic and social development and deep adjustment of industrial structure to: accelerate the construction of a clean, low-carbon, safe and efficient energy system, and the construction of a low-carbon transportation system; improve the low-carbon development quality both in urban and rural areas; strengthen the promotion and application of green and low-carbon major scientific and technological achievements; continue to consolidate and enhance the capacity of carbon sinks; improve the level of green and low-carbon development of openness to the outside world; and improve laws and regulations, standards and statistical monitoring systems and other ways to achieve carbon peak and neutrality goals [10]. In October 2021, the State Council issued the Action Plan for Carbon Peak by 2030. This provides a general plan for the promotion of carbon peak during the 14th Five-Year Plan (2021–2025) and the 15th Five-Year Plan (2026–2030), and it focuses on the deployment of the Ten Actions for Carbon Peak. It is clearly emphasized that carbon peak should be included throughout the whole process and all aspects of economic and social development [11], covering energy, industry, transportation, transport, science and technology, finance and other related key areas and industries.

The establishment of the 1 + N policy system has integrated a number of key areas and industries, enabling relevant departments to formulate supporting policies around the realization of carbon peak and neutrality goals. The carbon emissions trading market alone cannot effectively regulate small- and medium-sized greenhouse gas emitters outside the trading market. Therefore, in the process of controlling greenhouse gas emissions and achieving energy conservation and emission reduction, China has consciously introduced various regulatory tools, such as information regulation, market regulation, administrative regulation and economic regulation to achieve carbon peak and neutrality goals, and has harvested certain results in greenhouse gas emission reduction. The 1 + N policy system makes full use of economic regulatory tools, such as carbon taxes and financial subsidies, which can compensate, to a certain extent, for the shortcomings of the carbon trading market and, thus, help achieve carbon peak and neutrality goals [12].

### 2.3. Environmental Protection Law, Climate Change Law, Energy Law and Other Related Legal Systems

In addition to the above-mentioned specific laws, regulations and policies, the environmental protection law, climate change law, energy law and other related legal systems in China also provide measures for issues related to the process of achieving carbon peak and neutrality goals. In the legal system of environmental protection, China has formed a system of specialized environmental legislation, with the Environmental Protection Law as the backbone, consisting of individual laws, such as the Law on Prevention and Control of Atmospheric Pollution, the Law on Promotion of Cleaner Production, the Law on Promotion of Circular Economy and the Law on Environmental Impact Assessment [13]. On this basis, various departments of the State Council provide detailed regulations on specific issues through administrative regulations and departmental rules, such as Measures for the Registration of Pollutant Emissions Declaration and Measures for the Administration of Environmental Standards. The above-mentioned laws and regulations provide a legal guarantee for the realization of carbon peak and neutrality goals by promoting environmental protection and preventing air pollution, improving ecological environment and enhancing carbon sink capacity, and strictly protecting responsibilities and clarifying penalty mechanisms. It should be emphasized that the environmental protection law system contains a more mature environmental impact assessment mechanism, and actors must conduct environmental impact assessments in accordance with the law when preparing relevant development and utilization plans and constructing projects that have an impact on the environment. The system provides a good foundation for the next stage of incorporating greenhouse gas emissions into the environmental impact assessment mechanism through relevant regulations. Moreover, the environmental protection law system also establishes an incentive mechanism for environmental protection, rewarding units and individuals who have made significant achievements in protecting and improving the environment. In stimulating the development of low-carbon industries, government subsidies [14] and tax incentives [15] are the policy tools.

In the climate change legal system, China completed the Law on Climate Change Response (Draft) in 2014. This draft serves as the first law directly related to greenhouse gas control. It clearly proposes that in order to cope with climate change, China should adhere to energy conservation, update the energy structure, optimize the industrial structure, develop a low-carbon economy, strengthen ecological protection and construction and strengthen publicity and education, as well as promoting international cooperation with scientific and technological progress and innovation as the support. However, for various reasons, the draft has not yet been formally promulgated and implemented. In addition, China amended the Meteorological Law in 2016, specifying that the legislative purpose of the law is to rationally develop, utilize and protect the climate. According to the provisions of the Meteorological Law, the competent meteorological agencies of the State Council shall monitor atmospheric components that may cause climate deterioration and shall conduct climate feasibility studies when carrying out urban planning, national key construction projects and major regional economic development projects. The above-mentioned articles regulate the obligations of state agencies and units, including the government, to control greenhouse gas emissions from the perspective of climate protection.

In the energy legal system, the Energy Conservation Law and the Renewable Energy Law clearly propose to strengthen the management of energy use, and China should reduce energy consumption, reduce pollutant emissions and stop energy waste in all aspects, from energy production to consumption. The control of greenhouse gas emissions and the achievement of carbon peak and neutrality goals are also supported and guaranteed by the above-mentioned laws. In addition, the Coal Law, the Electricity Law, the Measures for the Administration of Coal Production Licenses, the Rules for the Implementation of the Measures for the Administration of Coal Production Licenses, the Regulations on Foreign Cooperation in the Exploitation of Onshore Petroleum Resources, the Regulations on Foreign Cooperation in the Exploitation of Offshore Petroleum Resources, the Regulations on the Supervision and Administration of the Safety of Civil Nuclear Facilities and other energy-related laws and regulations stipulate that in the process of rational development and utilization of energy, environmental-protection-related laws and regulations must be observed. By regulating energy production and consumption, greenhouse gas emissions can be strictly controlled, which is of direct importance to achieve carbon peak and neutrality goals [16].

## 3. Characteristics of the Legal System in China to Achieve Carbon Peak and Neutrality Goals

As mentioned before, China is an early participant in international climate change governance and works with the international community to establish a universal platform for all countries to communicate and cooperate on the issue of controlling carbon emissions [4]. While implementing international treaties and strengthening international cooperation, the legal guarantee to achieve carbon peak and neutrality goals in China shows profound Chinese characteristics. From the perspective of China itself, whether in the legislative field, the executive field or the judicial field, the leading ideas, institutional structure and operational mechanisms of the legal system to achieve carbon peak and neutrality goals reflect the uniqueness of China’s special economic system, political system and social culture.

### 3.1. A New National Legal System with Concentrated Efforts for Carbon Peak and Neutrality Goals

A distinctive feature of the legal system for carbon peak and neutrality goals is the construction of the new national system with concentrated efforts, combining the role of the government with market mechanisms. The Action Plan for Carbon Neutrality by 2030 clearly identifies the construction of a new national system as an important principle to achieve carbon neutrality. In contrast to the separation of powers in the West, China has established a democratic centralism that emphasizes the important role of government power in national development and social change. The reliance on government power in China’s development process is influenced by two main factors. One factor is the historical role of the former Soviet political system, but the influence of that factor is gradually diminishing. The other factor is the real needs of China as a developing country. Through the skewed allocation of resources by government power, China has succeeded in a number of major scientific and technological challenges and in the construction of livelihood protection projects, and has, thus, developed a path of dependence on the power of a strong executive.

In the process of reducing carbon emissions, the Chinese administration uses a hierarchical organizational structure to distribute the task of reducing carbon emissions to all levels of government and to various functional departments. In 2022, seven national ministries and commissions, including the Ministry of Ecology and Environment, jointly formulated the Implementation Plan for the Synergy of Pollution and Carbon Reduction, which incorporates the completion of control and reduction for greenhouse gas emissions into ecology and environment-related assessments on the basis of a clear division of responsibilities among individual units. The assessment of quantitative indicators for officials is considered to be central to China’s goal of environmental governance [17]. In addition, the central administration has made a significant financial commitment to reducing carbon emissions. Taking the clean winter heating pilot in Northern China carried out since 2017 as an example, the central administration provided financial support for pilot cities to promote clean ways of heating instead of bulk coal burning for heating. As of December 2021, a total of 63 pilot cities have been listed in the pilot program. Based on the full allocation of subsidy funds during the three-year demonstration period, the four batches of pilot cities will need to allocate a total of RMB 76.2 billion in subsidy funds. In fact, in the context of China’s legal normative system, the aforementioned normative documents formulated by the executive authorities to promote the achievement of carbon peak and neutrality goals are not laws in a narrow sense but public policies.

The new national system mainly means the participation of market mechanisms. In the Kyoto Protocol, achieving emission reduction targets through market mechanisms has become an important pathway [18]. Unlike relying solely on planned economic instruments to achieve public goals, the socialist market economy system established since China’s reform and opening up has become an important institutional background for the current promotion of carbon peak and neutrality goals. In 2011, China launched carbon emissions trading pilots in seven provinces and cities, and planned to achieve national carbon emissions trading by 2021. The country not only specifies carbon emission rights in the law, but also establishes a trading system for buying, selling and mortgaging carbon emission rights through centralized bidding and agreement. The carbon emission trading market system regards carbon emission allowances as a new type of asset in addition to cash assets, physical assets and intangible assets, and provides incentives for parties to seek more cost-effective ways to reduce emissions, so as to achieve total control and dynamic balance of carbon emissions [19].

In the process of promoting carbon emission reduction in China, the two instruments, government role and market mechanism, have different weights in different institutional environments. In the legal system of carbon emission trading, it mainly follows the principle of market-led as the main body and administrative supervision as the guarantee [20], and the government maintains the order of the trading market by establishing carbon emission quotas, risk management of trading and information disclosure of trading. In addition, China combines carbon emission reduction with industrial structure optimization and scientific and technological innovation, and tends to play the role of policy incentives based on the market system, such as financial support for the development of green and low-carbon industries, increased government procurement of green and low-carbon products and the implementation of tax incentives for new energy and clean energy vehicles and vessels. In the legal system of carbon emission regulation, carbon emission testing and evaluation of key industries and fields, carbon emission pollution damage assessment and supervision and inspection of production operators mainly rely on the government.

### 3.2. Central and Local Initiative in the Legal System for Carbon Peak and Neutrality Goals

From the perspective of national governance, the process of legal development in China to reduce carbon emissions can be viewed from two perspectives—at the central government level and at the local government level. How to arouse the governance effectiveness of local governments is an important test of the governance capacity in a unitary state. In 1956, Chairman Mao argued that the country’s development required both central and local activism, requiring both the central government to set unified rules of action and local governments to exercise activism in the context of local conditions [21]. This view has influenced institutional design and policy implementation in China. The development process of the legal system for carbon peak and neutrality goals is based on the interaction between the central government and local governments [22].

The central government, on behalf of the country, actively fulfills its carbon reduction obligations in international treaties and provides the following three main types of unified orders. (1) It provides a unified value and ideological foundation. Sustainable development is an important way for developing countries to achieve the two daunting goals of economic development and carbon emission reduction in a coordinated manner [23]. The inclusion of ecological civilization in the Constitution in 2018 aims to make ecological protection the purpose of social development and to raise awareness of environmental protection in society as a fundamental law. Central government documents also repeatedly state that lucid waters and lush mountains are invaluable assets, which considers economic development and environmental protection as two things that complement each other. The central government’s assessment of local governments no longer focuses only on economic indicators, but also incorporates the ecological benefits and sustainability of economic development into the evaluation system. (2) It provides uniform carbon emission targets and standards. In addition to the overall targets of achieving carbon peak by 2030 and carbon neutrality by 2060, the central government has set some more detailed data targets. The Outline of the 14th Five-Year Plan (2021–2025) and Vision 2035 of the National Economic and Social Development of the People’s Republic of China, released in 2021, proposes that: during the 14th Five-Year Plan period, the proportion of non-fossil energy in total energy consumption will increase to about 20%, and energy consumption and carbon dioxide emissions per unit of GDP will be reduced by 13.5% and 18%, respectively. The Action Plan for Carbon Peak by 2030 further proposes that, by 2030, the proportion of non-fossil energy consumption will reach about 25%, and carbon dioxide emissions per unit of GDP will drop by more than 65% compared to 2005, so as to successfully achieve the goal of carbon peak by 2030. (3) It provides a unified carbon emission trading market. The carbon emission trading market in China is mainly divided into two levels. The primary market mainly includes the total amount setting and initial allocation of carbon emission allowances, while the secondary market includes trading venues and trading behaviors for both supply and demand sides. Through the control of total carbon emissions and the allocation of allowances to provinces and municipalities, the national goals of carbon peak and carbon neutrality will be achieved. However, due to the incipient system, China’s carbon emissions trading has experienced problems, such as fragmented allocation of emission trading allowances, unclear legal attributes of carbon emission allowances and even inconsistent rules for allowance allocation [24].

Local governments also play an important role in the task of reducing carbon emissions. In federal countries, such as the United States, the main issue facing the carbon reduction mandate is how to align states under a national environmental protection goal and increase collaboration among states [25]. The problem for China is how to mobilize local initiatives. In addition to the local environmental governance assessment tasks assigned by central government, local governments themselves also have an inherent need to enhance investment attractiveness and residents’ happiness through environmental protection. China’s Legislative Law makes special provisions for local legislation, especially for the People’s Congresses and governments of municipalities with subordinate districts to legislate on matters of environmental protection, giving them room for institutional innovation. As can be seen from Table 1, all eight provinces and municipalities in China’s carbon emissions trading pilot projects have introduced specific legislation on the management of carbon emissions trading. For example, five provinces and cities, including Shenzhen, Fujian, Guangdong, Hubei and Shanghai, legislate in the form of local government regulations, while Tianjin, Beijing and Chongqing legislate in the form of regulatory documents. In addition, Shenzhen Special Economic Zone has formulated local regulations, and Shaanxi, Shenyang and Xinyu have also regulated carbon emissions trading by formulating regulatory documents.

## 4. The Improvement Direction and Outlook of the Legal System to Achieve Carbon Peak and Neutrality Goals in China

The framework of the legal system established in China, with reductions in carbon emissions as its core, provides an important guarantee for the achievement of carbon peak and neutrality goals. Its consistency with international common practice and, at the same time, its integration with China’s politics, economy and culture have revealed some problems in the process of promoting the implementation of the legal system, which may affect the effectiveness of the legal guarantee.

From the perspective of domestic law, the levels of economic development and greenhouse gas emissions vary greatly from region to region in China [26], and the construction of a legal guarantee system for carbon peak and neutrality goals should focus on the relationship between different regions and subjects. First, China should enact unified legislation at the national level as soon as possible to establish the basic principles and legal rules that should be followed to reduce carbon emissions. At present, there are many differences and disagreements in the exploration of the system of carbon emission trading in different places, which is not conducive to the formation of a unified carbon emission trading market, and the basic contents, such as the standard of setting the total amount of carbon emission and the principles and ways of allocating carbon emission allowances, should be stipulated through national unified legislation as soon as possible. Second, China should continue to encourage localities, under the guidance of national unified legislation and based on the actual economic and social development of the region, to build a local rule of law guarantee path for energy conservation and emission reduction through local regulations and local government regulations, so as to unify the universality and specificity [7] of carbon reduction efforts in all regions of the country in the process of achieving carbon peak and neutrality goals. Third, China should strengthen the role of social groups and organizations in the process of reducing carbon emissions, mobilize social forces to join the carbon peak and neutrality actions, encourage the private sector to share project data, information and technology, and avoid prematurely transferring the risk of responsibility to local governments and the general public [27].

From the perspective of international law, the achievement of carbon peak and neutrality goals is a systemic project faced by all of humanity. Greenhouse gas regulation needs to emphasize cooperation and collective action [28], and no single individual, region or country can achieve this task alone. China should continue to adhere to the principle of common but differentiated responsibilities and the spirit of territorial equity, further strengthen international cooperation, promote internal and external coordination and provide a more solid legal guarantee for the overall achievement of carbon peak and neutrality goals. First, China should effectively fulfill its international treaty obligations, promote the harmonization of domestic legal norms with international treaty obligations and promote the interface between the domestic rule of law system and the international governance system in the process of actively revising relevant laws, regulations and policies and urgently introducing specialized legal norms. Second, China should strengthen international exchanges and cooperation, actively participate in the development of global carbon emission reduction standards, promote the improvement of the United Nations Framework Convention on Climate Change system, complete the negotiation of the implementation rules of the Paris Agreement and provide practical international standards for carbon peak and carbon neutrality goals. Third, under the Belt and Road initiative, China should establish a multilateral carbon emission reduction mechanism, realize low-carbon interconnection, strengthen green economic and trade cooperation and build green economic and trade partnerships, so as to promote the development of more fair and reasonable global carbon emission reduction standards and cooperation models, and provide a more favorable international governance environment to achieve carbon peak and neutrality goals [29].

## 5. Conclusions

China is the largest developing country in the world, which means achieving carbon peak and neutrality goals is both a challenge and an opportunity. On the one hand, China must meet the survival and development needs of its 1.4 billion people, and the complete abandonment of high-energy-consuming and high-emission industries in a short period of time will affect the employment of workers and social stability [30]. On the other hand, the elimination of high-energy-consuming and high-emission industries is an inevitable direction for China to optimize its industrial structure and achieve high-quality social development.

In order to achieve the goal of carbon peak and carbon neutrality, China initially established a unified legal system by formulating special laws and establishing the “1 + N” policy system in accordance with international treaty obligations and domestic needs. However, according to the realization process of carbon peak and carbon neutralization, establishment of the legal system is still in the initial stage and it is necessary to continuously improve the legalization of carbon emission management from both domestic and foreign levels. At the domestic level, China should continue to give full play to the advantages of the “the new national system with concentrated efforts and resources “, and on the premise of a unified legislation system of the central, local governments should be encouraged to explore the legal path to manage the reduction in and neutralization of local carbon emission based on regional economic and social development status. At the international level, to adhere to the spirit of a community with shared future for mankind, China should deeply participate international frameworks, such as South–South cooperation, the Shanghai Cooperation Organization and the Belt and Road Initiative, with an effort to explore the potential development path of more equitable global carbon emission reduction standards and models, and take an initiative in the global climate governance to address ongoing global climate change.

China’s determination to reduce carbon emissions is reflected in the act of pushing toward its carbon peak and neutrality goals through a complex legal system. Its practice and experience in the construction of the legal system for carbon peak and neutrality goals can also serve as a reference for other countries to achieve their carbon emission reduction targets.

## Figures and Tables

**Table 1:** Current legislation on carbon emissions trading management in major provinces and cities.

*No.*	*Legislation Name*	*Implementation Time*	*Legislation Authority*	*Legislation Level*
1	Shenzhen Carbon Emissions Trading Management Measures	1 July 2022	Shenzhen Municipal People’s Government	Local Government Regulation
2	Shaanxi Province Carbon Emission Trading Management Implementation Rules (for trial implementation)	30 June 2022	Department of Ecology and Environment of Shaanxi Province	Local Regulatory Document
3	Shenyang Carbon Emission Trading Management Measures	1 September 2021	Shenyang Municipal People’s Government	Local Regulatory Document
4	Fujian Province Interim Measures for the Management of Carbon Emission Trading	7 August 2020	Fujian Provincial People’s Government	Local Government Regulation
5	Tianjin Interim Measures for the Management of Carbon Emission Trading	1 July 2020	Tianjin Municipal People’s Government	Local Regulatory Document
6	Guangdong Province Trial Measures for Carbon Emission Management	12 May 2020	Guangdong Provincial People’s Government	Local Government Regulation
7	Certain Provisions on Carbon Emission Management in Shenzhen Special Economic Zone	5 September 2019	Shenzhen Municipal People’s Congress	Local Government Regulation
8	Interim Measures for Carbon Emission Management and Trading in Hubei Province	1 November 2016	Hubei Provincial People’s Government	Local Government Regulation
9	Xinyu Carbon Emissions Trading Management Measures	25 July 2014	Xinyu Municipal People’s Government	Local Regulatory Document
10	Beijing Carbon Emission Trading Management Measures (for Trial Implementation)	28 May 2014	Beijing Municipal People’s Government	Local Regulatory Document
11	Interim Measures for the Management of Carbon Emission Trading in Chongqing	26 April 2014	Chongqing Municipal People’s Government	Local Regulatory Document
12	Interim Measures for the Management of Carbon Emission Trading in Shanghai	20 November 2013	Shanghai Municipal People’s Government	Local Government Regulation

## Data Availability

Not applicable.
